# Spontaneous Coronary Artery Dissection in a Transplanted Heart

**DOI:** 10.1016/j.jaccas.2022.06.016

**Published:** 2022-11-02

**Authors:** Taher Sbitli, Bdoor Bamousa, Jehad Alburaiki, Mosaad Alhussein, Ali Almasood

**Affiliations:** a Department of Medicine, Alfaisal University, Riyadh, Saudi Arabia; b Heart Center, King Faisal Specialist Hospital & Research Center, Riyadh, Saudi Arabia; c King Saud bin Abdulaziz University for Health Sciences, Riyadh, Saudi Arabia

**Keywords:** acute coronary syndrome, cardiac transplant, dissection, myocardial infarction, ACS, acute coronary syndrome, EMB, endomyocardial biopsy, FMD, fibromuscular dysplasia, LAD, left anterior descending, LHC, left-sided heart catheterization, PCI, percutaneous coronary intervention, RHC, right-sided heart catheterization, SCAD, spontaneous coronary artery dissection

## Abstract

We report the case of a 37-year-old man who presented with shortness of breath 1 year post heart transplantation. He was receiving tacrolimus, methylprednisolone, and mycophenolate. An angiogram showed spontaneous coronary artery dissection involving the left anterior descending artery. Percutaneous coronary intervention was performed successfully, with stent placement and return of flow. (**Level of Difficulty: Advanced.**)

## Central Illustration

**Figure:**
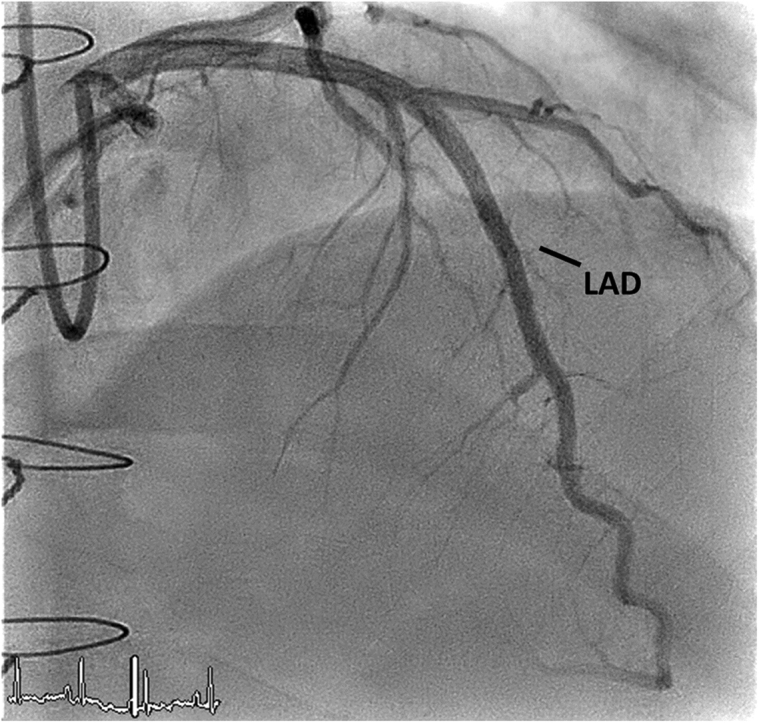

## History of Presentation

A 37-year-old man 1-year post-heart transplantation for idiopathic dilated cardiomyopathy presented to the clinic with symptoms of new onset exertional dyspnea and episodic palpitations associated with dizziness. The patient was afebrile and had no chest pain. His blood pressure of was 122/72 mm Hg, and his heart rate was 102 beats/min. Findings of cardiovascular and pulmonary examinations were normal.

**Learning Objectives**

• To recognize SCAD as a cause of ACS.
• To explore the possible role immunosuppressive therapy plays in the pathophysiology of SCAD.

## Past Medical History

**Figure 1:**
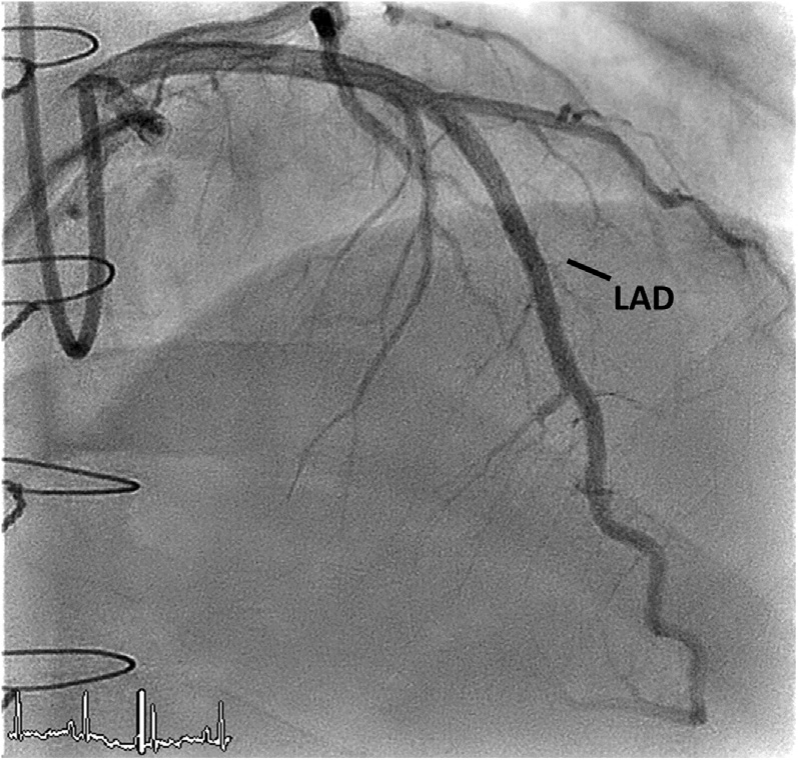
Left-Sided Heart Angiogram (Right Anterior Oblique Cranial View) This image obtained 2 months after heart transplantation shows normal flow. LAD = left anterior descending coronary artery.

He had a history of dilated cardiomyopathy that was treated with guideline-directed medical therapy and implantable cardioverter-defibrillator insertion, with subsequent cadaveric orthotopic heart transplantation from a healthy middle-aged man who had died of a subdural hematoma because of a road traffic accident. A post-transplant echocardiogram showed normal left ventricular systolic function with an ejection fraction of >55%. Two months after heart transplantation, a coronary angiogram with intravascular ultrasound was performed as baseline surveillance for cardiac allograft vasculopathy and showed normal flow in all vessels, with a normal intimal thickness (Figure 1). He was stable on maintenance immunosuppressive therapy with tapered prednisone (5 mg once daily), tacrolimus (4 mg twice daily, to achieve target levels according to protocol), and mycophenolate (1 g twice daily). Scheduled follow-ups showed a stable course with a normal echocardiogram 2 months before his presentation (Video 1).

## Differential Diagnosis

The differential diagnosis included transplant rejection (cellular or antibody-mediated), cardiac allograft vasculopathy, and graft dysfunction.

## Investigations

**Figure 2:**
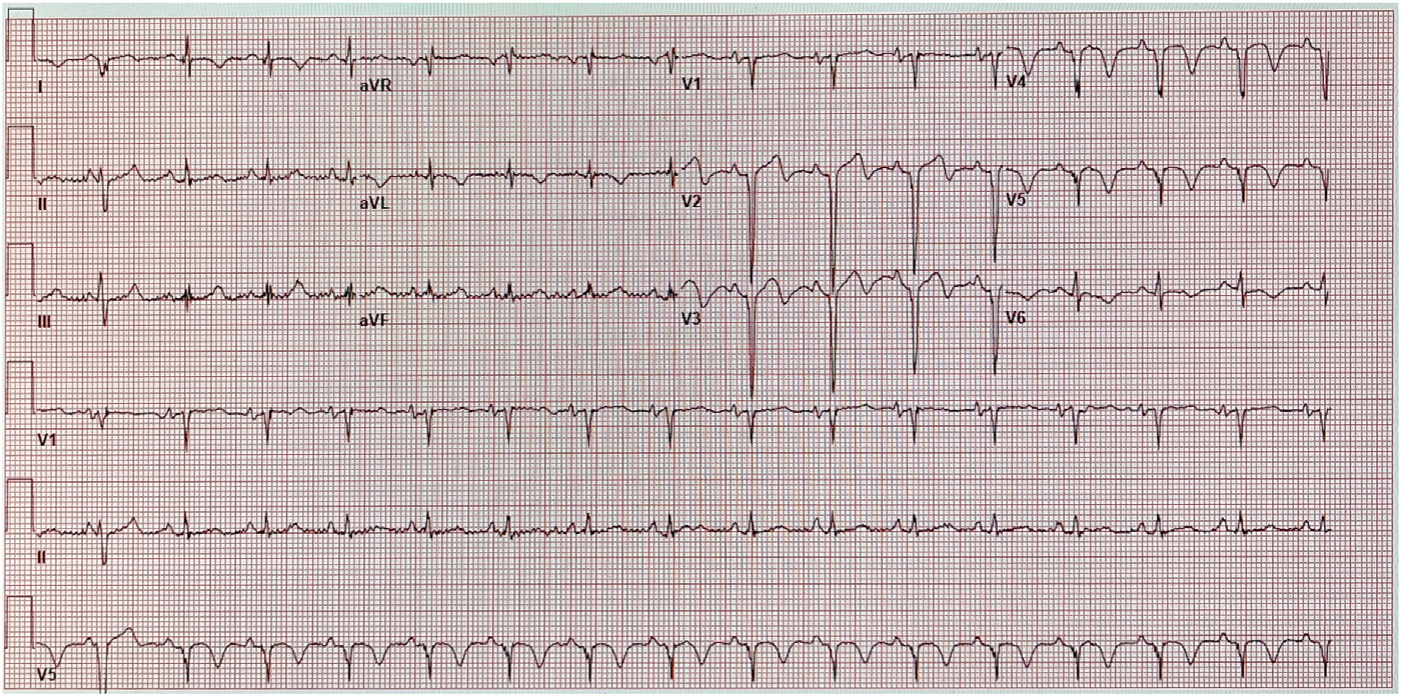
Electrocardiogram on Presentation The changes shown in the anterior leads are consistent with a left anterior descending artery infarct.

**Figure 3:**
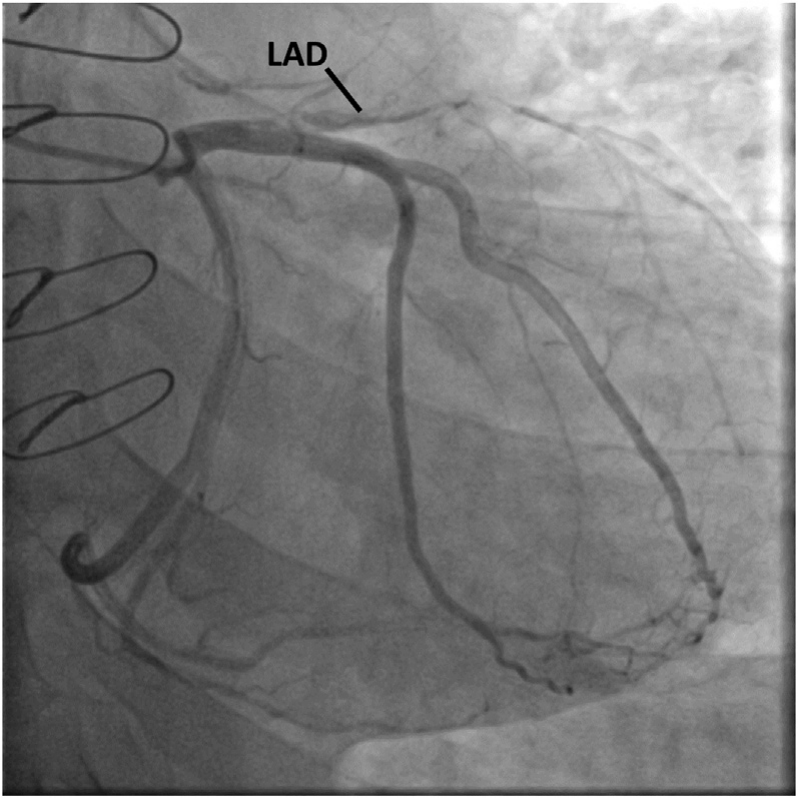
Left-Sided Heart Angiogram (Anteroposterior Caudal View) The image shows total occlusion of the left anterior descending coronary artery (LAD) from the ostium.

Blood laboratory work-up showed an elevated troponin value of 18 ng/L (normal <15 ng/L). The electrocardiogram showed ST-segment elevation and pathologic Q waves in the anterior leads consistent with an anterior myocardial infarction (Figure 2). A transthoracic echocardiogram demonstrated significant changes (Video 2): reduced left ventricular systolic function with an ejection fraction of 35% to 40%, regional wall motion abnormalities consistent with left anterior descending (LAD) coronary artery infarction, reduced right ventricular systolic function, and a moderately sized laminated apical thrombus. Right-sided heart catheterization (RHC) with endomyocardial biopsy (EMB) and left-sided heart catheterization (LHC) were performed. RHC showed normal hemodynamics and normal filling pressures. LHC revealed a spiral dissection resulting in total occlusion of the LAD artery lumen (Figure 3, Video 3). EMB showed no signs of rejection.

## Management

**Figure 4:**
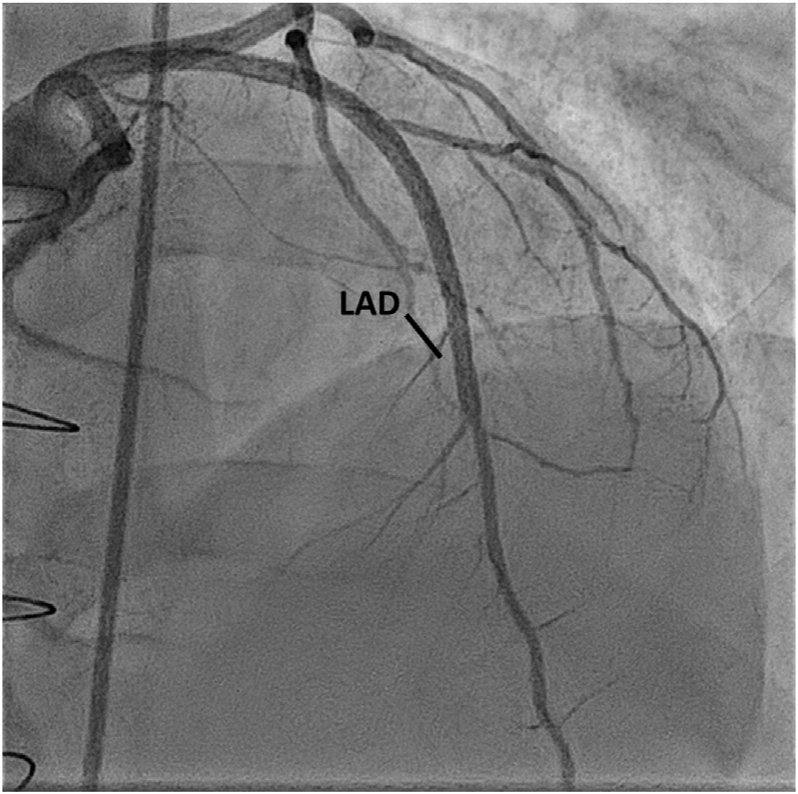
Left-Sided Heart Angiogram (Right Anterior Oblique Caudal View) The image shows return of flow post percutaneous coronary intervention. LAD = left anterior descending coronary artery.

The patient was started on heparin for the left ventricular thrombus, in addition to his regular medications. Percutaneous coronary intervention (PCI) with drug-eluting stent placement to the LAD artery was successfully done for the involved segment, with Thrombolysis In Myocardial Infarction flow grade 3 and a good angiographic result (Figure 4, Video 4). He was started on clopidogrel, aspirin, and warfarin. He improved clinically and was discharged with scheduled follow-ups.

## Discussion

First reported in 1931, spontaneous coronary artery dissection (SCAD) has been regarded as a rare disease with a prevalence ranging from 0.49% to 4% among patients with acute coronary syndrome (ACS).^1^^,^^2^ However, the prevalence of SCAD in the general population is yet to be determined. Women are the most affected, accounting for up to 84% of cases.^3^

Although the pathophysiology of SCAD is unclear, it is theorized to have 2 possible mechanisms: 1) an intimal dissection resulting in blood flow between the intima and media, causing a false lumen; and 2) rupture of the vasa vasorum leading to accumulation of hematoma within the medial space.^4^

Multiple risk factors have been associated with an increased occurrence of SCAD. Underlying arteriopathy is often the predisposing factor. Of the arteriopathies, fibromuscular dysplasia (FMD) is by far the most commonly reported association, with a prevalence up to 86%. Screening of patients for FMD is not yet widely recommended.^5^ Chronic systemic inflammatory diseases are present in 8.9% of patients and may have a potential association, with chronic inflammatory vasculitis rather than acute inflammation being the most probable link.^6^ The evidence for this association is lacking, as most are case reports.

There have been a few cases of SCAD in liver and kidney transplant recipients.^7^, ^8^, ^9^ The use of immunosuppressant agents such as calcineurin inhibitors and high-dose steroids is a common factor in these cases. Although associations have been reported, there is a lack of supportive data. The multiple theories of how calcineurin inhibitors can affect the arterial walls include stimulation of vasoconstriction, production of reactive oxygen species, and increased sympathetic tone.^10^^,^^11^ Our patient was taking tacrolimus, which may possess some of the effects mentioned previously, although to a lesser extent compared with other calcineurin inhibitors.^7^ As far as we know, this is the second reported case of SCAD in a heart transplant recipient,^12^ and it is the second reported case with concurrent use of tacrolimus.^7^

Although management is still undetermined because of the lack of randomized controlled trials compared with atherosclerotic causes of ACS, conservative treatment is preferred on the basis of expert recommendations, if the coronary flow is normal. However, in patients with evidence of ischemia, as in our case, PCI should be considered for revascularization.^6^ Regular follow-ups should be scheduled because the rate of recurrence at 3 years post first SCAD is approximately 10.4%.^13^

## Follow-Up

At the clinic appointment 4 months after discharge, the patient had clinical improvement with no chest pain or shortness of breath. An echocardiogram showed a left ventricular ejection fraction of 45% to 50%, mild akinesia involving the distal LAD territory (Video 5), and normal valvular movement.

## Conclusions

There should be a high clinical suspicion for SCAD in patients presenting with ACS. On the basis of observations in our case and other published cases, the relationship between immunosuppressants and SCAD requires further research to improve care in patients with transplants and positively alter clinical outcomes of management. Some high-risk patients with SCAD should be managed with revascularization.

## Funding Support and Author Disclosures

The authors have reported that they have no relationships relevant to the contents of this paper to disclose.

## Appendix

Supplemental Video 1 Echocardiogram (Short-Axis View). Showing post-transplant normal left ventricular contraction.

Supplemental Video 2 Echocardiogram (Short-Axis View). Showing anterior septal hypokinesia consistent with left anterior descending artery infarct.

Supplemental Video 3 Left-Sided Heart Angiogram (Anteroposterior Caudal View). Total occlusion of the left anterior descending artery from the ostium.

Supplemental Video 4 Left-Sided Heart Angiogram (Left Anterior Oblique Caudal View). Return of flow post percutaneous coronary intervention.

Supplemental Video 5 Echocardiogram (Short-Axis View). Showing remnant anterior hypokinesia on follow-up.

## References

[1] Krittanawong C., Kumar A., Virk H.U.H. (2018). Trends in incidence, characteristics, and in-hospital outcomes of patients presenting with spontaneous coronary artery dissection (from a national population-based cohort study between 2004 and 2015). Am J Cardiol.

[2] Nishiguchi T., Tanaka A., Ozaki Y. (2016). Prevalence of spontaneous coronary artery dissection in patients with acute coronary syndrome. Eur Heart J Acute Cardiovasc Care.

[3] Franke K.B., Nerlekar N., Marshall H. (2021). Systematic review and meta-analysis of the clinical characteristics and outcomes of spontanous coronary artery dissection. Int J Cardiol.

[4] Naderi S. (2018). Spontaneous coronary artery dissection: an overview. Curr Atheroscler Rep.

[5] Franke K.B., Wong D.T.L., Baumann A. (2019). Current state-of-play in spontaneous coronary artery dissection. Cardiovasc Diagn Ther.

[6] Saw J., Mancini G.B.J., Humphries K.H. (2016). Contemporary review on spontaneous coronary artery dissection. J Am Coll Cardiol.

[7] Mallon D.H., McKenzie D., Dayer M. (2012). A spontaneous coronary arterial dissection associated with a calcineurin inhibitor. BMJ Case Rep.

[8] Verlaeckt E., Van de Bruaene L., Coeman M. (2019). Spontaneous coronary artery dissection in a patient with hereditary polycystic kidney disease and a recent liver transplant: a case report. Eur Heart J Case Rep.

[9] Tsimikas S., Giordano F.J., Tarazi R.Y. (1999). Spontaneous coronary artery dissection in patients with renal transplantation. J Invasive Cardiol.

[10] Seibert F., Behrendt C., Schmidt S. (2011). Differential effects of cyclosporine and tacrolimus on arterial function. Transpl Int.

[11] Trapp A., Weis M. (2005). The impact of immunosuppression on endothelial function. J Cardiovasc Pharmacol.

[12] Theertham A., Niazi K., Moreyra A. (2020). Spontaneous coronary artery dissection in heart transplant recipient. J Am Coll Cardiol.

[13] Saw J., Humphries K., Aymong E. (2017). Spontaneous coronary artery dissection: clinical outcomes and risk of recurrence. J Am Coll Cardiol.

